# Low-level laser therapy for the treatment of oral manifestations in a patient with toxic epidermal necrolysis: a case report^؆^

**DOI:** 10.1016/j.abd.2025.501234

**Published:** 2025-11-03

**Authors:** Victor Meza, Isidora Mujica, Marianne Kolbach, Claudio Escanilla, Fernando Valenzuela

**Affiliations:** a Department of Dermatology, Faculty of Medicine, Universidad de los Andes, Santiago, Chile; b Department of Oral Pathology, Faculty of Dentistry, Universidad de los Andes, Santiago, Chile; c Department of Dermatology, Clínica Universidad de los Andes, Santiago, Chile; d Department of Dermatology, Faculty of Medicine, University of Chile, Santiago, Chile

Dear Editor,

Toxic epidermal necrolysis (TEN) is an acute, potentially life-threatening mucocutaneous condition characterized by epidermal necrosis and detachment in response to primarily pharmacological triggers.^1^, ^2^, ^3^

Although more than 90% of patients with TEN present with oral mucosa involvement, evidence-based treatment protocols for these manifestations are still lacking, given the low incidence of this disease.^1^

We report the case of an adult patient with TEN, in which we used low-level laser therapy (LLLT) for his refractory-to-treatment oral manifestations.

A 38-year-old man with a history of bariatric surgery and depression, receiving chronic treatment with aripiprazole, escitalopram, esomeprazole and multivitamins; and starting modafinil, trazodone and acetazolamide within the past month, was referred to our department with a 5-day history of asthenia, unmeasured fever, conjunctivitis, anorexia and dysphagia. Generalized skin lesions subsequently appeared.

Upon admission, he was febrile (39 °C), tachycardic (110 bpm), and tachypneic, requiring orotracheal intubation for mechanical ventilation.

Physical examination revealed a generalized vesiculobullous rash involving more than 60% of the body surface area, along with skin denudation on the back and genitals, and hemorrhagic cheilitis. Nikolsky’s sign was positive.

A skin biopsy was performed, and histopathology revealed total epidermal necrosis, with a mild superficial perivascular lymphocyte infiltrate and negative direct immunofluorescence.

Additional laboratory tests ruled out infectious and autoimmune diseases.

TEN (SCORTEN scale: 3-points) was diagnosed, and multidisciplinary management was established, including withdrawal of non-essential drugs and cutaneous-systemic support measures. Causality assessment tools did not allow us to determine the culprit drug, given the patient’s polypharmacy and similar treatment initiation period.

Pulse methylprednisolone (500 mg/day for 3-days, with subsequent tapering) and intravenous immunoglobulin (2 g/kg over 3-days) were initiated. Serial skin and blood cultures were also performed, with early initiation of antibiotic therapy planned in case of superinfection.

Due to persistent clinical activity, a single dose of Etanercept 50 mg subcutaneously was prescribed on the fifth day of hospitalization.

The patient subsequently presented a favorable clinical cutaneous response, but with persistent hemorrhagic cheilitis and oral ulcers (Fig. 1). Therefore, on the eleventh day of hospitalization, PCR tests for Herpes Simplex Virus I/II and *Mycoplasma pneumoniae* on the oral mucosa were repeated, which were negative, and it was decided to initiate LLLT.

**Figure 1:**
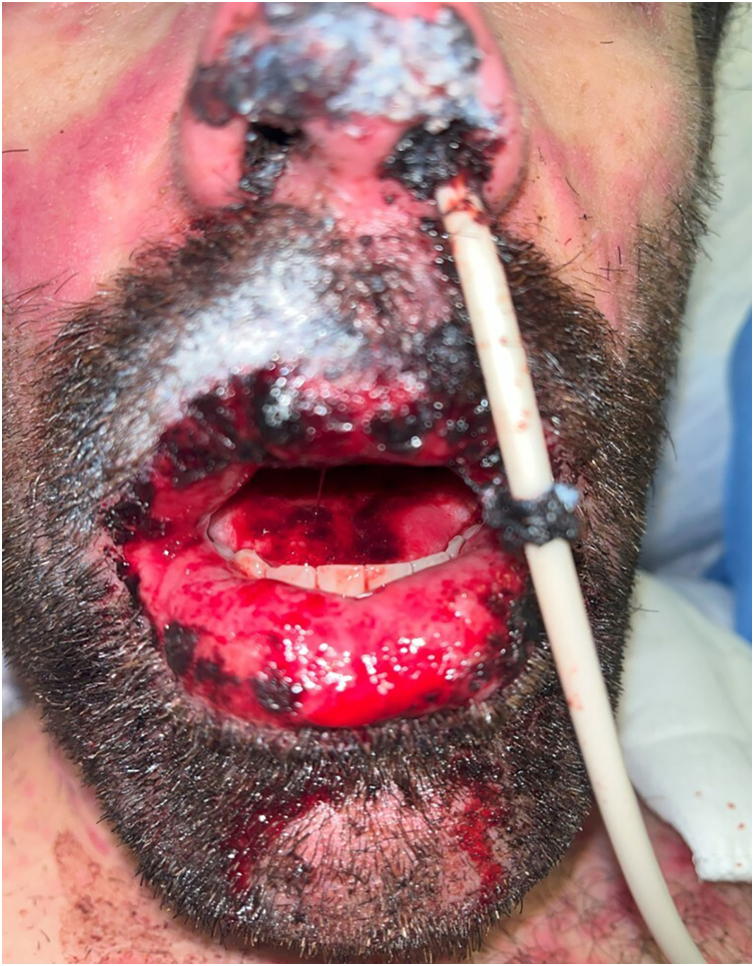
Hemorrhagic cheilitis and tongue ulcers, prior to the initiation of low-level laser therapy.

Using the *Laser Duo* device (InGaAIP, MMOptics), daily LLLT sessions were performed, with wavelengths of 660 nm and 808 nm, and a fluence of 6 J/cm^2^ per application point, including lips, jugal mucosa, hard palate, and tongue (Fig. 2).

**Figure 2:**
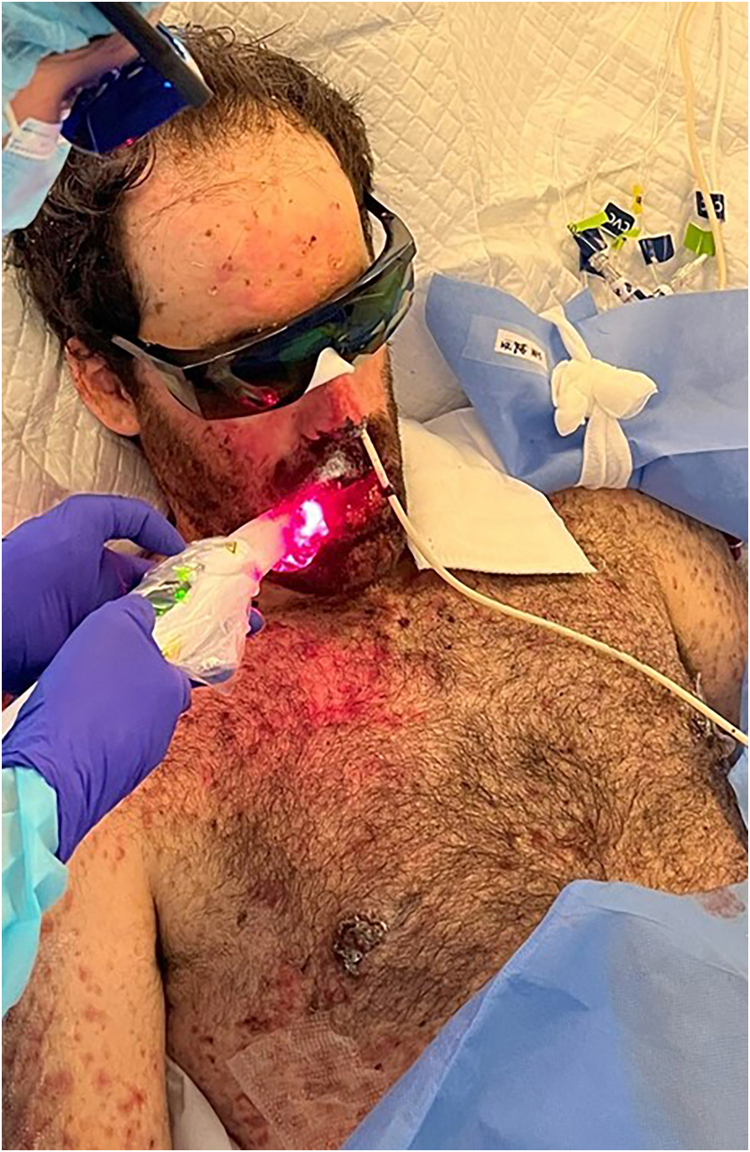
Application of low-level laser therapy. With the *Duo Laser* device (InGaAIP, MMOptics), daily sessions were carried out, with wavelengths of 660 nm and 808 nm, and a fluence of 6 J/cm^2^ per application point, including lips, jugal mucosa, hard palate and tongue.

A total of 6 sessions were completed, which were subsequently discontinued due to the favorable clinical response (Fig. 3A‒C), assessed as pain reduction and resolution of the hemorrhagic cheilitis.

**Figure 3:**
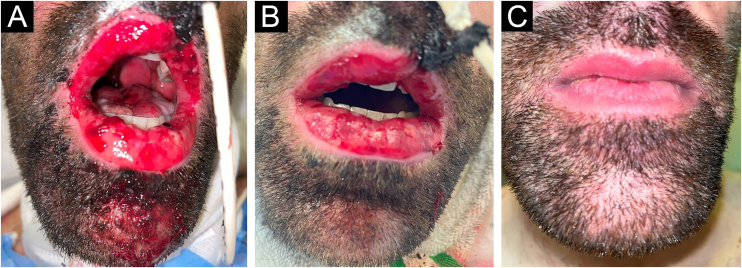
(A‒C) Clinical response of the oral mucosa. (A) 48 hours from the start of Low-Level Laser Therapy (LLLT). (B) 72 hours from the start of LLLT. (C) 1-week after hospital discharge (23-days from the first LLLT session).

The patient was finally discharged on his 26^th^ day of hospitalization, with no complications resulting from LLLT up to the current 6-month follow-up.

LLLT is a noninvasive light therapy that does not involve thermal or ablative phenomena and, as its name suggests, consists of the administration of low-density energy.^4^

Its mechanism of action is based on photobiomodulation, a process in which the interaction of photons with mitochondrial cytochromes and porphyrins produces a temporary release of nitric oxide, stimulating the synthesis of adenosine triphosphate and promoting the formation of reactive oxygen species, resulting in cell activation.^4^, ^5^ Successively, various intracellular pathways that modulate the synthesis of proteins and nucleic acids are triggered; the levels of cytokines and inflammatory mediators are regulated; and cell differentiation and proliferation are promoted.^5^, ^6^

Several studies have linked LLLT with analgesic, anti-inflammatory, and restorative properties,^4^, ^5^ which have promoted its use in various conditions, including those with mucosal involvement, such as oral lichen planus, mucous membrane pemphigoid, recurrent aphthous stomatitis, and mucositis secondary to chemotherapy, among others.^5^, ^7^, ^8^, ^9^

To our knowledge, there are only 3 previous reports of its use in cases of serious adverse drug reactions, including TEN (all in pediatric patients), with favorable clinical outcomes, as in the case of our patient.^1^, ^2^, ^3^

As a limitation of the case presented, it is not possible to rule out a potential late benefit of systemic therapies in our patient’s oral manifestations, which may have been complementary or overlapping with the action of LLLT. Furthermore, guidelines on the optimal parameters for the use of LLLT are still lacking.

In conclusion, LLLT may represent a valid therapy for refractory oral manifestations in patients with adverse drug reactions like TEN, given its analgesic, anti-inflammatory, and restorative properties. Nonetheless, randomized trials to support these findings are warranted.

## ORCID ID

Isidora Mujica: 0000-0001-9923-8390

Marianne Kolbach: 0009-0002-1465-1085

Claudio Escanilla: 0000-0002-8399-2708

Fernando Valenzuela: 0000-0003-1032-9347

## Financial support

The authors declare that they have not received any type of financial support.

## Authors' contributions

Victor Meza: Study conception and planning; critical literature review; preparation and writing of the manuscript; data collection, analysis and interpretation; manuscript critical review.

Isidora Mujica: Study conception and planning; preparation and writing of the manuscript; analysis and interpretation; manuscript critical review.

Marianne Kolbach: Preparation and writing of the manuscript; analysis and interpretation; manuscript critical review.

Claudio Escanilla: Preparation and writing of the manuscript; analysis and interpretation; manuscript critical review.

Fernando Valenzuela: Study conception and planning; preparation and writing of the manuscript; analysis and interpretation; manuscript critical review; manuscript critical review.

## Research data availability

Does not apply.

## Conflicts of interest

None declared.
